# Observations on the Constitutions, According to the Classification of Recamier

**Published:** 1845-09

**Authors:** Charles B. Voigt


### THE MEDICAL EXAMINER,

AND

### RECORD OF MEDICAL SCIENCE.

NEW SERIES—No. IX.—SEPTEMBER, 1845.

---

### ORIGINAL COMMUNICATIONS.

*Observations on the Constitutions, according to the classification of Recamier.* By Charles B. Voigt, M.D.

The expression constitution, as it occurs in common use, is often employed with reference to the powers and condition of the system collectively contemplated, as when a debilitated and cachectic state is spoken of as a vitiated or bad constitution. It is also frequently used with particular reference to the vital powers alone, as a strong or feeble constitution, and the like. Without intention of opposing these appropriations of the term, the meanings of which may be sufficiently manifest, at least in the connections in which they occur, the object of the following remarks is to invite attention to the subject of the constitutions in a stricter understanding of the term than applies to it in such and similar acceptations.

I allude to the constitutions as they are classified by Recamier and adverted to in the Manual of Pathology by Martinet.* The reference which is made to them in the work in question is brief and of a very general character; but as they would seem, as distinguished by the classification of these authorities, to be founded in nature, and to involve points of practical interest, they would appear entitled to more attention than I am aware of their having usually attracted.

*Page 29.

To exhibit the views of the authorities above referred to on this subject, I will quote the passage from the Manual of Pathology in which they are presented. It is as follows:—"A knowledge of the constitutions will enable us to foresee in a great measure the form which diseases are likely to assume, and the course they will probably run. According to Professor Recamier, constitutions may be divided into the active, passive, ataxic, and refractory. Observation has shown that in persons who present the characters of the active constitution, namely, those whose functions and actions are performed with energy and regularity, the return to health is more prompt and easy, and their diseases are more regular and less fatal, if properly treated from the commencement; that in those of a passive constitution, whose functions and actions are feeble, slow and dull, though still regular, diseases are tedious in their progress, and tardy in their return to health, and consequently have a tendency to remain stationary; that in those, whether active or passive, who are of an ataxic habit, that is, who exhibit in their different vital phenomena, any incoherence, irregularity, or confusion, diseases will present similar characters, will arise from apparently insufficient causes, and often assume such a formidable character as to render it impossible to arrest their progress; lastly, in persons whose constitutions are such as to merit the appellation of refractory, that is, who manifest a certain energy in their functions, with considerable resistance in their disturbance, disease when once excited presents a similar tenacity, and generally resists every method of treatment."

It will be perceived that the constitutions in this passage are based upon certain characters of "functions and actions," and "vital phenomena," which are represented as peculiar in particular individuals, and as exerting special influences upon the phenomena and progress of disease. Admitting the constitutions as they are here contemplated and distinguished, observation leads me to refer their basis more explicitly to three general properties of the organic system, or at least of near alliance to the vascular system. With a view to brevity, they may be succinctly specified in this place. They are, 1st, Irritability of the organic system, or the susceptibility to organic impressions. 2dly, The mode of action of the vascular system, or the peculiar tendency or determination of action which the latter is liable to assume under the perturbations of disease* 3dly, The tone,

*In one class of persons a peculiar tendency of determination towards the periphery will be observed; in another, the tendency of action is concentrative; and in a third it may be more peculiarly introversive or centripetal. These peculiarities of action are disclosed on the supervention of disturbances in the habitual equipoise of health.

strength, or power of the organic system. These properties are common to all individuals, but as they occur under peculiar and different characters in different classes of persons, a foundation is afforded for the distinction of different and particular constitutions.

In the above extract four forms of constitution are specified, the "active," "passive," "ataxic" and "refractory." Possibly there may be adequate grounds for this four-fold distinction; but so far as my own observations on this subject have extended, I can claim familiarity with three only, namely, the active, passive and refractory.

With ataxic phenomena I have seldom met, and consequently have but an imperfect practical acquaintance with them. In the few instances in which I have witnessed them, they appeared to complicate what I supposed ordinary examples of the active and refractory forms of constitution; and indeed in the above quoted passage, they are referred to and spoken of as a "habit," and as liable to occur in conjunction with the phenomena of the active and passive constitutions. Hence it might be observed that if they are not liable to be presented as an individual and fundamental type of organic action and independent of others, they can scarcely be considered entitled to be assumed as the basis of a particular constitution. But on the supposition that they are secondary or accessary phenomena, liable to complicate others of more steady and constant occurrence, they may be important, by combination with the latter, in establishing compound constitutions. On this view, if well founded, there might be an ataxo-active constitution, &c., but not a simple ataxic form.

Not disposed to underrate the importance of ataxic irregularities in disease, in which they are sometimes the most conspicuous or serious of the morbid disturbances,—for reasons above referred to, I shall omit their further consideration in the ensuing remarks, and depend upon the general allusion which they have thus incidentally received, to expose, in some measure, the connection which they sustain with this subject. Whether they should be ranked as the basis of a special constitution, or viewed only as an extraordinary element, occasionally compounding more constant and regular phenomena, may be left to further investigation to determine. Perhaps, in some instances, they may be more dependent upon particular morbid states and depravations, than on fixed and habitual dispositions of the organism. Perhaps, on the property of irritability, when existing in high development and unusual excitability; or finally, on the concurrence of both these conditions.

Omitting then the "ataxic" constitution, but three remain, namely, the passive, the active and the refractory, and assuming the three organic properties before referred to as their basis, I shall correspondently dispose and restrict the remainder of the subject.* These three forms of constitution appear well characterized and are generally easily recogaized.

### 1. *Of the Passive Constitution.*

Torpor or bluntness of organic sensibility is a prominent feature of this constitution. The organic actions are slow and unenergetic, and readily conform to therapeutic impressions. Inflammation is seldom intensely acute; generally of slow progress; and not prone very rapidly to eventuate in disorganization. Action tends to the periphery, though it is not vigorously supported in the direction which it affects. Hence it is liable to disturbances and inward and local accumulations; but as the system is unirritable, much acuteness of capillary action is not always induced by them, and the system will often right itself before material suffering has been sustained. Irritation occurring in this constitution, rather tends to assume a subacute or chronic form, and may endure for a considerable period without implicating very serious consequences upon the parts affected. Stimuli are better tolerated than in the other forms of constitution, and in moderate quantities are not very perturbating to the vascular system. It is an organization which is easily tranquillized when the subject of disease, and for the most part soon relieved by therapeutic means. It is centrifugal in its mode of action; ductile under treatment; and easily exhausted.

### 2. *Of the Active Constitution.*

This form of constitution is distinguished by susceptibility to organic impressions and mobility of nervous and vascular action. Disease evinces a higher grade of acuteness than in the preceding, and is liable to involve more serious consequences by protracted duration. The actions of the system may be very easily and promptly roused, and in disease may be very readily over-excited. In febrile cases, in particular, collapse, or complete introversion of action is sometimes suddenly witnessed in the active constitution as a result of treatment unduly potent or stimulating. Congestions are prone to occur.

Two or three classes and some varieties of this constitution are distinguishable in practice. In one the organic powers are easily exhausted; in another they are more tenacious and strong; and in a third irritability is more particularly conspicuous. Ex-

*A separate and detailed account of these organic properties has been omitted. They are specially alluded to under the several constitutions.

citability of organic action, correspondently modified by these peculiarities, is the character of all. The temperaments* probably contribute to the particular modifications of this and other forms of constitution.

### 3. *Of the Refractory Constitution.*

As the epithet imports, this form of constitution evinces a decidedly untoward or intractable tendency in the character of its actions. Even in the ordinary state of health, some degree of excitation or irritation is liable to exist upon some of the membranes, organs, or internal surfaces, and to correspondently affect and disturb the organs and functions associated with the seat of disorder. When the subject of acute disease, the latter usually assumes the highest grade of intensity, and is peculiarly difficult of reduction and control. It tends to active effusions and modifications of structure, or to settle in a confirmed chronic state. It presents the firmly contracted or wire-like pulse, implicates the severest suffering, and evolves a crowd of violent symptoms. Instead of the centrifugal tendency, vascular action evinces a decided disposition to local concentration and excess, and as most frequently observed, upon locations verging towards the axis of the system.

Irritability or organic sensibility is very conspicuous. It exhibits, in this constitution, its maximum of intensity and keenest susceptibility. If it be unduly excited in disease, it reacts upon and is sure to aggravate a local affection. The increment of excitement or activity imparted to the system, instead of taking a centrifugal direction, appears to bend itself upon the local disease, and to produce an evident aggravation of its condition. As developed in this constitution, this property is cognizable by its susceptibility to impressions, and extreme intolerance of excitation in disease.

Organic strength is vigorous, and endurance great.†

The subject may be concluded by some brief remarks, founded upon the peculiarities which distinguish the particular forms of constitution which have been adverted to in the preceding descriptions.

The lowness of irritability, moderate or feeble organic power, and tendency to centrifugal action, indicate, in the passive constitution, that depletion and the antiphlogistic system should be

*The temperaments, it might be remarked, refer to the organic systems or tissues; the constitutions, as here understood, to organic properties.

†The above applies to a strongly characterized example of the refractory constitution.

cautiously imposed. On account of the lowness of irritability, stimuli, with a view to an alterative action, when required, may be exhibited at earlier periods, and with less liability to mischief, than in cases in which other forms of constitution or different conditions of the organic properties obtain; and as the centrifugal determination is the habitual tendency, the latter class of remedies often proves highly beneficial in the treatment of its disease.* The feebler forms of inflammation, which disease often assumes in this constitution, frequently admit of the employment of tonics, such, even, as quinine, and the preparations of iron. To operate favourably, however, capillary action should be benignly and equably improved by them.†

In the active constitution the organic properties present a different aspect. Irritablity is acute, vascular action excitable and easily disturbed, and the organic powers inclining to vigour. In inflammatory disease, local capillary action is easily enhanced, and, particularly in fevers, introverted action or collapse is very liable to be induced by impressions of an inappropriate or unduly potent character. Its powers being often strong and capable of considerable endurance, depletion more liberal than in the passive constitution, or decidedly free, is compatible with it, and often indispensable in the management of its inflammatory and congestive disease. The pulse instead of being depressed under this treatment, will generally rise, and vascular action become much more open and free. The same tenor of remark is also applicable in reference to the employment of other antiphlogistic treatment.

As a general principle, the acuteness of irritability, which is incident to this constitution, is opposed to perturbating remedies in the active and acute forms of disease. To conform to the peculiarities and influences of this property, alterative impressions should be gentle, and, when resorted to, reference should be had to the existing state of the organic powers; if the latter are vigorous or firm, there is danger of imparting too strong an impression, and superinducing the consequences of the latter, which as previously intimated are often very serious. The action of alteratives, particularly when the organic powers retain some degree

*The determination to the periphery when not dependent on febrile action, is well known to be highly conducive to the relief, particularly, of internal disease. If it be not the essential condition of an equalized capillary action throughout the system, the latter probably always implicates it to a certain degree.

†When the actions of the system are equalized by articles which appear to accomplish this object principally by favourably interesting the capillaries, the pulse will often manifest a slight degree of tenseness, an effect apparently ascribable to an impression transmitted to the heart and arteries from the whole capillary system.

of energy, should be especially addressed to the capillary system.

In the refractory constitution, the organic powers are strong, irritability intense, and the tendency of action strongly concentrative. Hence, in the management of its diseases, which correspond in severity with the forces and irritability of this peculiarity of system, depletion and reduction are of primary importance; by these means only, and the abstraction of every cause of vigor and excitement, can the peculiar intensity of its morbid actions be overcome. As a general remark, the irritability and concentrative tendency of action, which are manifested by it, particularly in its more decided examples, are most effectually controlled when the system is reduced to a state of debility. In this condition also, attempts at equalizing capillary action are most successfully instituted. Active stimulants and tonics—such of the latter especially as enhance nervous energy without correspondently equalizing capillary action, (their usual mode of action when they disagree,) may be pronounced incompatible with the characters of this constitution when under the influence of febrile and inflammatory disease.

